# Prognostic value of PD-L1 and Siglec-15 expression in patients with nasopharyngeal carcinoma

**DOI:** 10.1038/s41598-022-13997-2

**Published:** 2022-06-21

**Authors:** Ju Zhao, Hanshan Yang, Hui Hu, Chao Liu, Min Wei, Yumei Zhao, Yudan Chen, Yongxia Cui, Ping Chen, Kang Xiong, Yun Lu, Hongru Yang, Linglin Yang

**Affiliations:** 1 Department of Geratology, The Luzhou People’s Hospital, Luzhou, 646000 China; 2 Department of Oncology, The Affiliated Hospital of Southwest Medical University, Luzhou, 646000 China; 3 Department of Emergency Medicine, The Affiliated Hospital of Southwest Medical University, Luzhou, 646000 China; 4 Department of Oncology, The Chengdu Seventh People’s Hospital, Chengdu, 610000 China; 5 Department of Health Management, The Affiliated Hospital of Southwest Medical University, Luzhou, 646000 China; 6 Department of Oncology, The Third Hospital of Mianyang, Mianyang, 621000 China

**Keywords:** Cancer, Immunology, Diseases, Oncology

## Abstract

Sialic acid-binding immunoglobulin-like lectin 15 (Siglec-15) might be involved in the activation of important pathways related to tumor immune escape, along with programmed death-ligand 1 (PD-L1). Here, we aimed to investigate the correlation between the expression of Siglec-15 and PD-L1 in nasopharyngeal carcinoma (NPC) patients. We determined the expression of PD-L1 via immunohistochemical staining and that of Siglec-15 via immunofluorescence staining in 182 NPC tissue samples. A significant correlation was identified between the PD-L1 and Siglec-15 expression (P = 0.000). Moreover, Kaplan–Meier survival curves showed that PD-L1 expression was associated with improved overall survival (OS) (P = 0.025) and Siglec-15 expression was associated with improved distant failure-free survival (D-FFS) (P = 0.048). Moreover, multivariate Cox analysis showed that PD-L1 and Siglec-15 were independent predictors of OS (P = 0.020) and D-FFS (P = 0.047), respectively. The results of the log-rank test and Cox regression analyses showed that patients exhibiting no PD-L1/Siglec-15 expression had significant advantages regarding OS, compared to other groups (P = 0.037). PD-L1 and Siglec-15 may represent novel biomarkers for predicting the prognosis of NPC patients. Siglec-15 may be considered as a potential target for the development of therapeutics for NPC treatment in the future.

## Introduction

Nasopharyngeal carcinoma (NPC) is prevalent in East and South Asia. In 2018, approximately 129,000 new cases of NPC, which account for merely 0.7% of all cancers, were reported worldwide^1^. The distribution of NPC cases varied significantly with ethnicity and region as well as genetic makeup and environment, and an incidence rate of up to 70% was observed in Southern Asia^2^. In most patients, metastasis to the lymph node or other organs had already occurred during initial diagnosis. Significant progress has been achieved in the treatment of NPC with the development of intensity-modulated radiotherapy (IMRT), but the local recurrence and distant metastasis rates after treatment are still more than 30%^2^. It is known that the survival and prognosis of patients with NPC are affected by several factors, such as the TNM stage, TIGAR, TC3B, and other biological factors^3,4^. The identification of more effective biomarkers not only facilitates prognosis prediction, but also provides promising targets for treatment purposes, which would be extremely useful for NPC patients.

In recent years, immunotherapy has successfully risen to the forefront and become a frequently used method for cancer treatment, and is the fourth treatment modality to be used in addition to surgery, chemotherapy, and radiotherapy^5^. Programmed death-ligand 1 (PD-L1) inhibitors have shown good efficacy for NPC treatment^6–8^. In addition, PD-L1 molecules expressed on the surface of tumor cells can predict the curative effect and survival prognosis^9^. Lee et al. believe that NPC patients with high expression of PD-L1 have longer disease-free survival after treatment^10^, but other studies suggest that patients with high expression of PD-L1 have shorter disease-free survival, shorter overall survival, and poor prognosis^11–13^. Meta-analysis showed that high PD-L1 expression predicts a shorter OS in NPC patients^14^. In contrast, the results of another study showed that the differences between PD-L1 expression, OS, and disease-free survival were not statistically significant^15^. The association between PD-L1 expression in NPC patients and tumor development and prognosis is still controversial.

Although anti-PD-1/PD-L1 therapy is currently the treatment of choice and clinically effective immunotherapy treatment available for cancer, only 20% to 30% of human solid tumors respond to anti-PD-1/PD-L1 therapy^16–18^. The reduced effects of anti-PD-1/PD-L1 therapy suggest that the possibility of developing new immunotherapeutic drugs targeting other potential immune blockade pathways, such as Siglec-15, discovered in the latest study, needs to be explored further^19^. The monoclonal antibody to Siglec-15 (α-S15) has exhibited good efficacy in vivo, and the first phase I/II clinical trial (NCT03665285) of the Siglec-15 humanized monoclonal antibody (NC318), used for the treatment of several solid malignancies, such as uterine, lung, and head and neck cancers, is nearing completion^20^. Professor Chen’s protein sequence analysis shows that the extracellular domain of Siglec-15 includes the immunoglobulin variable region (IgV) and type 2 constant region (IgC2), which exhibits a homology of > 30% with the B7 gene family, of which PD-L1 is a member. This indicates that Siglec-15 has a close relationship with PD-L1^19^. Immunofluorescence staining results of 241 patients with non-small cell lung cancer (NSCLC) showed that there was a negative relationship between expression levels of PD-L1 and Siglec-15 in the tumor tissues of patients with NSCLC^19^. In addition, Kaplan–Meier survival analysis results derived from the TCGA database showed that the survival rate of lung cancer patients showing Siglec-15 overexpression was significantly decreased in the progression-free survival period, suggesting that Siglec-15 can be used as an important biomarker of prognosis^21^. Fudaba et al. showed that the detection of Siglec-15 in patients with primary central nervous system (CNS) lymphoma was indicative of a good prognosis^22^. However, the relationship between Siglec-15 expression and the prognosis of NPC patients has not yet been identified.

In this study, we analyzed the expression of Siglec-15 and PD-L1 using immunohistological assays. We aimed to evaluate whether the expression of Siglec-15 and PD-L1 could be used to predict outcomes in NPC patients in the most efficient manner.

## Results

### Patient characteristics

In our study, 182 NPC patients were enrolled, including 128 males and 54 females. The age of enrolled patients was 20–73 years, and the median age was 48 years. Forty-eight patients (26.4%) had an initial diagnosis of stage I–II, and 134 patients (73.6%) were initially diagnosed at stage III–IV. The Union for International Cancer Control/American Joint Committee on Cancer (UICC/AJCC) T stage was identified to be T1–2 for 97 patients (53.3%) and T3–4 for 85 patients (46.7%). Lymph node metastases were absent in 33 patients (18.1%) and present in 149 patients (81.9%). Using the WHO pathology classification system, we identified that 4 (2.2%), 65 (35.7%), and 113 (62.1%) out of 182 patients had grade I, grade II, and grade III disease. The data are presented in Table 1.

**Table 1:** Relationship between Siglec-15 and PD-L1 expression and clinicopathologic variables in nasopharyngeal carcinoma.

Variables	Number	PD-L1		P-value	Number	Siglec-15		P-value
		n (+)	n (−)			n (+)	n (−)	
**Gender**								
Male	128	88 (68.7%)	40 (31.3%)	0.473	128	54 (42.2%)	74 (57.8%)	0.067
Female	54	40 (74.1%)	14 (25.9%)		54	15 (27.8%)	39 (72.3%)	
**Age (years)**								
≤ 48	96	66 (68.7%)	30 (31.3%)	0.622	96	36 (37.5%)	60 (62.5%)	0.904
> 48	86	62 (72.1%)	24 (27.9%)		86	33 (38.4%)	53 (61.6%)	
**WHO pathology classification**								
I (keratinizing)	4	4 (100%)	0 (0%)	0.156	4	1 (25.0%)	3 (75.0%)	0.386
II (non-keratinizing)	63	48 (76.2%)	15 (23.8%)		63	28 (44.4%)	359 (55.6%)	
III (undifferentiated)	115	76 (66.1%)	39 (33.9%)		115	40 (34.8%)	75 (65.2%)	
**T stage**								
T1–2	97	62 (63.9%)	35 (36.1%)	0.043*	97	39 (40.2%)	58 (59.8%)	0.496
T3–4	85	66 (77.6%)	19 (22.4%)		85	30 (35.3%)	55 (64.7%)	
**Lymph node metastasis**								
N0	33	26 (78.8%)	7 (21.2%)	0.240	33	9 (27.3%)	24 (72.7%)	0.164
N1–3	149	102 (68.5%)	47 (31.5%)		149	60 (40.3%)	89 (59.8%)	
**TNM stage**								
I–II	48	30 (62.5%)	18 (37.5%)	0.166	48	16 (33.3%)	32 (66.7%)	0.446
III–IV	134	98 (73.1%)	36 (26.8%)		134	53 (39.6%)	81 (60.4%)	

*Siglec-15* Sialic acid-binding immunoglobulin-like lectin 15; *PD-L1* programmed cell death-Ligand 1.

The bold value represents p > 0.05, indicating that the difference is statistically significant.

### Relationship between PD-L1 and Siglec-15 expression and clinicopathological characteristics

**Figure 1:**
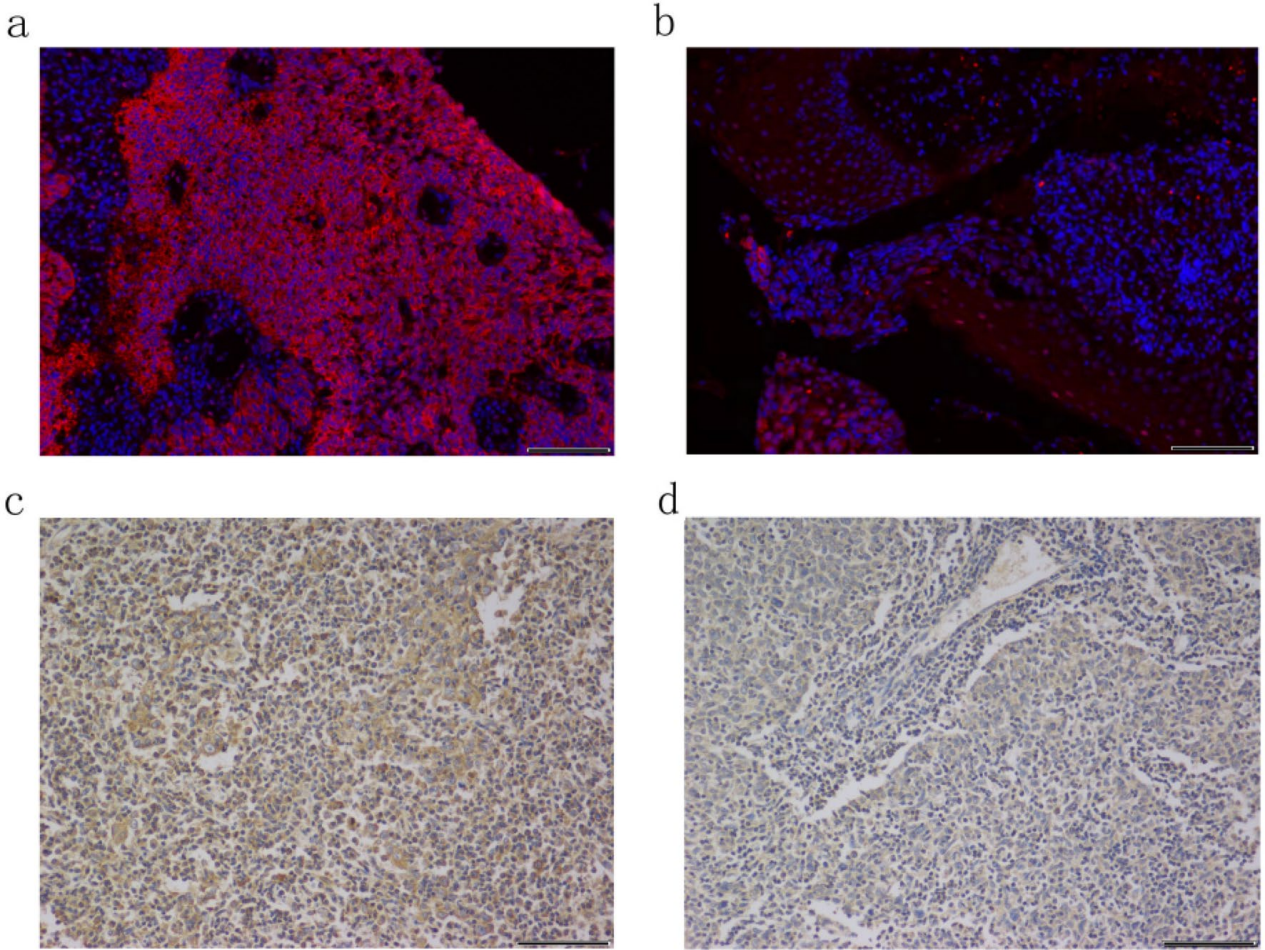
Immunostaining of PD-L1 and Siglec-15 protein expression in nasopharyngeal carcinoma. (**a)** Siglec-15+  (× 200); (**b)** Siglec-15– (× 200); (**c**) PD-L1+  (× 200); (**d**) PD-L1− (× 200).

Cell membrane staining was performed for the detection of both PD-L1 and Siglec-15. Tumors of 128 patients were PD-L1+  (70.3%), whereas 54 (29.7%) were PD-L1−. Sixty-nine Siglec-15+  (37.9%) and 113 Siglec-15− (62.1%) tumors (Table 2, Fig. 1) were observed. In addition, Chi-squared tests were applied to detect the relevance between PD-L1 or Siglec-15 and clinicopathological parameters, such as age, sex, pathology classification, TNM stage, and lymph node metastasis. However, the correlation between Siglec-15 and clinicopathological factors was not statistically significant. Similarly, except for the TNM stage, there was no other relevant factors with PD-L1 (Table 1).

**Table 2:** The association between Siglec-15 and PD-L1 expression.

	PD-L1		Total	R	P-value
	+	−			
**Siglec-15**					
+	36	33	69	− 0.311	0.000
−	92	21	113		
Total	128	54	182		

*Siglec-15* Sialic acid-binding immunoglobulin-like lectin 15, *PD-L1* programmed cell death-Ligand 1.

### Correlation between PD-L1 and Siglec-15 expression in 56 NPC patients

Spearman’s correlation analysis was performed to analyze the association between the expression of PD-L1 and Siglec-15. The expression of PD-L1 was significantly and inversely correlated with that of Siglec-15 (R =  − 0.311, *P* = 0.000, Table 2), and 36, 33, 92, and 21 NPC patients presented with PD-L1+/Siglec-15+, PD-L1−/Siglec-15 + , PD-L1+/Siglec-15−, and PD-L1−/Siglec-15− tumors, respectively (Table 2).

### Association of PD-L1 and Siglec-15 expression with clinical outcomes

**Figure 2:**
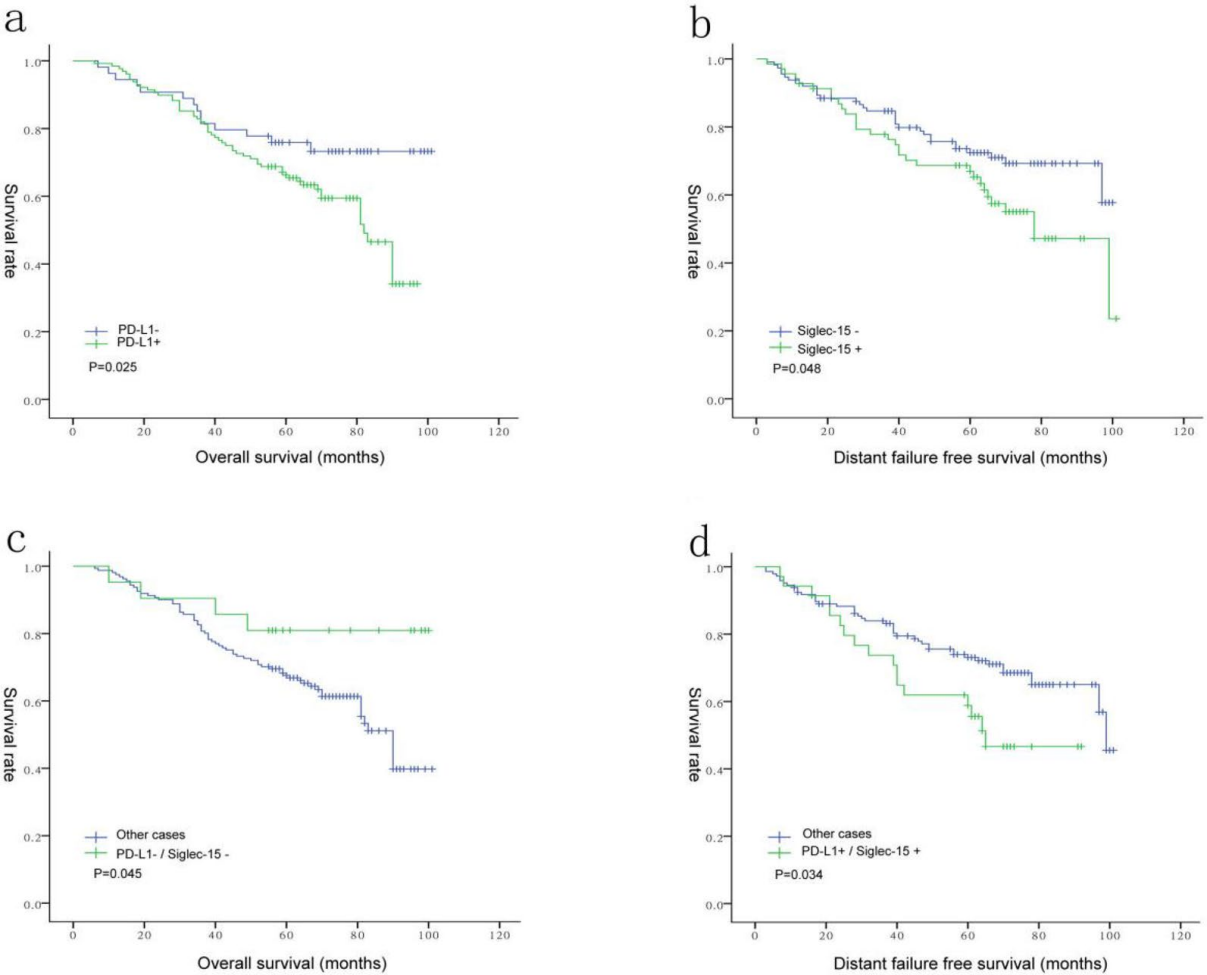
(**a**) Correlation between PD-L1 expression and overall survival (OS); (**b**) correlation between Siglec-15 expression and D-FFS; (**c**) correlation between PD-L1 and Siglec-15 expression and OS; (**d**) correlation between PD-L1 and Siglec-15 expression and D-FFS.

Our study focused on the expression of PD-L1 and Siglec-15 in NPC patients, therefore, single and multiple comparisons of OS, failure-free survival (FFS), distant failure-free survival (D-FFS), and local regional failure-free survival (LR-FFS) rates were performed under different conditions. As shown in Fig. 2A, NPC patients with PD-L1+ tumors had a less optimal OS (*P* = 0.025, Table 3), compared to patients with PD-L1− tumors, as revealed by Kaplan–Meier survival analysis and the log-rank test. There was a decreasing trend between LR-FFS and the two previously mentioned factors, although the data are not statistically different (Table 3). Similarly, patients with Siglec-15+ tumors had relatively poor D-FFS (*P* = 0.048, Table 3) rates compared to patients with Siglec-15− tumors (Fig. 2B).

**Table 3:** The association between clinicopathological variables and disease outcome.

Variables	OS	P-value	LR-FFS	P-value	D-FFS	P-value	FFS	P-value
	n (%)		n (%)		n (%)		n (%)	
**Gender**								
Male	79/128 (61.7%)	0.809	89/128 (69.5%)	0.235	79/128 (61.7%)	0.178	74/128 (57.8%)	0.321
Female	31/54 (57.4%)		43/54 (79.6%)		40/54 (74.1%)		36/54 (66.7%)	
**Age (years)**								
≤ 48	65/96 (67.7%)	0.022*	72/96 (76.0%)	0.279	66/96 (64.6%)	0.201	60/96 (62.5%)	0.337
> 48	45/86 (52.3%)		60/86 (73.3%)		53/86 (59.3%)		50/86 (58.1%)	
**WHO pathology classification**								
I (keratinizing)	4/4 (100%)	0.288	4/4 (100%)	0.464	4/4 (100%)	0.325	4/4 (100%)	0.289
II (non-keratinizing)	41/63 (65.2%)		46/63 (73.0%)		39/63 (66.1%)		39/63 (66.1%)	
III (undifferentiated)	65/115 (56.5%)		82/115 (71.3%)		76/115 (61.9%)		67/115 (61.9%)	
**T stage**								
T1–2	67/97 (69.1%)	0.032*	84/97 (86.6%)	0.000*	64/97 (66.0%)	0.697	63/97 (60.4%)	0.150
T3–4	43/85 (50.6%)		48/85 (56.5%)		55/85 (54.7%)		47/85 (54.7%)	
**Lymph node metastasis**								
N0	22/33 (66.7%)	0.433	25/33 (75.8%)	0.608	28/33 (84.8%)	0.015*	23/33 (69.7%)	0.204
N1–3	88/149 (59.1%)		107/149 (71.8%)		91/149 (61.1%)		87/149 (58.4%)	
**TNM stage**								
I–II	33/48 (68.8%)	0.460	43/48 (89.6%)	0.009*	33/48 (68.8%)	0.801	31/48 (64.6%)	0.697
III–IV	77/134 (57.5%)		89/134 (66.4%)		86/134 (64.2%)		79/134 (59.0%)	
**PD-L1 expression**								
(+)	70/128 (54.7%)	0.025*	88/128 (68.8%)	0.052	84/128 (65.6%)	0.593	77/128 (60.2%)	0.615
(−)	40/54 (74.1%)		44/54 (81.5%)		35/54 (64.8%)		33/54 (61.1%)	
**Siglec-15 expression**								
(+)	43/69 (62.3%)	0.821	51/69 (73.9%)	0.739	38/69 (55.1%)	0.048*	38/69 (63.7%)	0.324
(−)	67/113 (59.3%)		81/113 (71.7%)		81/113 (71.7%)		72/113 (55.1%)	
PD-L1 (+) Siglec-15 (−)/other case	50/92 (54.3%)	0.114	65/92 (70.7%)	0.471	66/92 (71.7%)	0.214	60/92 (65.2%)	0.349
	60/90 (66.7%)		67/90 (74.4%)		53/90 (58.9%)		50/90 (55.6%)	
PD-L1 (+) Siglec-15 (+)/other case	90/146 (61.6%)	0.398	109/146 (74.7%)	0.229	101/146 (69.2%)	0.034*	93/146 (63.7%)	0.090
	19/35 (54.3%)		23/35 (65.7%)		18/35 (51.4%)		17/35 (48.6)	
PD-L1 (−) Siglec-15 (−)/other case	93/161 (57.8%)	0.045*	116/161 (72.0%)	0.546	104/161 (64.6%)	0.31	98/161 (60.9%)	0.976
	17/21 (81.0%)		16/21 (76.2%)		15/21 (71.4%)		12/21 (57.1%)	
PD-L1 (−) Siglec-15 (+)/other case	87/149 (58.4%)	0.346	104/149 (69.8%)	0.077	99/149 (66.4%)	0.807	89/149 (59.7%)	0.572
	23/33 (69.7%)		28/33 (84.8%)		20/33 (60.6%)		21/33 (63.6%)	

*Siglec-15* Sialic acid-binding immunoglobulin-like lectin 15, *PD-L1* programmed cell death-Ligand 1, *WHO* World Health Organization, *OS* overall survival, *FFS* failure-free survival, *LR-FFS*, local regional failure-free survival, *D-FFS* distant failure-free survival.

The bold value represents p>0.05, indicating that the difference is statistically significant.

The correlation of clinicopathological variables conditions with OS and D-FFS was determined via Cox analyses (Tables 4, 5). PD-L1+ tumors were related to a poorer OS (*P* = 0.020, Table 4), but the differences in LR-FFS, D-FFS, and FFS (*P* > 0.05, respectively, not displayed) were not statistically significant. Values for PD-L1+ were constant in univariate and multivariate analyses. Similarly, Siglec-15 expression was associated with a poorer D-FFS (*P* = 0.047, Table 5) in univariate and multivariate analyses, but was not associated with OS, LR-FFS, or FFS (*P* > 0.05).

**Table 4:** Univariate and multivariate Cox proportional hazard analyses of clinicopathologic variables for the OS rate.

Variable		Univariate analysis			Multivariate analysis		
		HR	95% Confidence interval	P-value	HR	95% Confidence interval	P-value
Age (years)	≤ 48 vs > 48	0.941	0.573–1.545	0.811	0.882	0.520–1.498	0.642
Sex	Male vs Female	0.586	0.367–0.934	0.025*	0.592	0.364–0.964	0.035*
Pathology classification	I/II/III	1.226	0.742–2.027	0.427	1.308	0.764–2.239	0.328
T stage	T1–2 vs T3–4	0.602	0.376–0.965	0.035*	0.594	0.334–1.058	0.077
N stage	N0 vs N1–3	0.775	0.407–1.475	0.437	0.666	0.337–1.316	0.242
TNM stage	I–II vs III–IV	0.807	0.456–1.430	0.463	1.218	0.604–2.456	0.581
PD-L1 expression	+ vs −	0.520	0.290–0.933	0.028*	0.481	0.261–0.889	0.020*
Siglec-15 expression	+ vs −	1.057	0.653–1.710	0.822	0.901	0.544–1.494	0.687

*Siglec-15* Sialic acid-binding immunoglobulin-like lectin 15, *PD-L1* programmed cell death-Ligand 1, *WHO* World Health Organization, *OS* overall survival.

The bold value represents p>0.05, indicating that the difference is statistically significant.

**Table 5:** Univariate and multivariate Cox proportional hazard analyses of clinicopathologic variables for the D-FFS rate.

Variable		Univariate analysis			Multivariate analysis		
		HR	95% Confidence interval	P-value	HR	95% Confidence interval	P-value
Age (years)	≤ 48 vs > 48	1.498	0.827–2.716	0.183	1.253	0.672–2.335	0.478
Pathology classification	I/II/III	0.726	0.442–1.192	0.205	0.832	0.499–1.386	0.480
Sex	Male vs female	0.856	0.511–1.434	0.555	0.982	0.569–1.695	0.948
T stage	T1–2 vs T3–4	0.906	0.551–1.490	0.698	0.754	0.423–1.342	0.336
N stage	N0 vs N1–3	0.341	0.137–0.852	0.021*	0.324	0.127–0.832	0.019*
TNM stage	I–II vs III–IV	0.928	0.518–1.663	0.802	1.309	0.670–2.560	0.431
PD-L1 expression	+ vs −	0.860	0.495–1.497	0.595	0.662	0.366–1.197	0.172
Siglec-15 expression	+ vs −	0.611	0.373–1.002	0.051	0.590	0.351–0.993	0.047*

*Siglec-15* Sialic acid-binding immunoglobulin-like lectin 15, *PD-L1* programmed cell death-Ligand 1, *WHO* World Health Organization, *D-FFS* distant failure-free survival.

Significant values are in bold.

We also comparatively analyzed the potential relevance between the expression patterns for a combination of PD-L1 and Siglec-15 and patient prognosis. The patterns assessed included: PD-L1+/Siglec-15+, PD-L1−/Siglec-15+, PD-L1+/Siglec-15−, PD-L1−/Siglec-15−. The PD-L1−/Siglec-15− tumor subgroup (n = 35) had a favorable prognostic effect on the OS (*P* = 0.045, Table 6, Fig. 2C). In contrast, the PD-L1+/Siglec-15+  (n = 21) pattern was associated with poor D-FFS rates (*P* = 0.034, Table 7, Fig. 2D). No significant correlation was observed between the other patterns and the OS, LR-FFS, D-FFS, or FFS (*P* > 0.05).

**Table 6:** Univariate and multivariate Cox proportional hazard analyses of clinicopathologic variables for the OS rate.

Variable		Univariate analysis			Multivariate analysis		
		HR	95% Confidence interval	P-value	HR	95% Confidence interval	P-value
Age (years)	≤ 48 vs > 48	0.941	0.573–1.545	0.811	0.919	0.543–1.555	0.752
Sex	Male vs Female	0.586	0.367–0.934	0.025*	0.606	0.374–0.984	0.043*
Pathology classification	I/II/III	1.226	0.742–2.027	0.427	1.285	0.757–2.181	0.352
T stage	T1–2 vs T3–4	0.602	0.376–0.965	0.035*	0.556	0.312–0.992	0.047*
N stage	N0 vs N1–3	0.775	0.407–1.475	0.437	0.646	0.330–1.266	0.203
TNM stage	I–II vs III–IV	0.807	0.456–1.430	0.463	1.281	0.637–2.576	0.487
Correlation between PD-L1 and Siglec-15	PD-L1 (−) Siglec-15 (−)/other case	2.678	0.974–7.363	0.056	2.998	1.070–8.403	0.037*

*Siglec-15* Sialic acid-binding immunoglobulin-like lectin 15, *PD-L1* programmed cell death-Ligand 1, *WHO* World Health Organization, *OS* overall survival.

The bold value represents p>0.05, indicating that the difference is statistically significant.

**Table 7:** Univariate and multivariate Cox proportional hazard analyses of clinicopathologic variables for the D-FFS rate.

Variable		Univariate analysis			Multivariate analysis		
		HR	95% Confidence interval	P-value	HR	95% Confidence interval	P-value
Age (years)	≤ 48 vs > 48	1.498	0.872–2.716	0.183	1.193	0639–2.225	0.580
Sex	Male vs female	0.726	0.442–1.192	0.205	0.828	0.496–1.384	0.472
Pathology classification	I/II/III	0.856	0.511–1.434	0.555	0.947	0.548–1.634	0.844
T stage	T1–2 vs T3–4	0.906	0.551–1.490	0.698	0.764	0.427–1.366	0.363
N stage	N0 vs N1–3	0.341	0.137–0.852	0.021*	0.335	0.130–0.859	0.023*
TNM stage	I–II vs III–IV	0.928	0.518–1.663	0.802	1.262	0.644–2.472	0.497
Correlation between PD-L1 and Siglec-15	PD-L1 (+) Siglec-15 (+)/other case	0.550	0.313–0.967	0.038*	0.590	0.331–1.052	0.074

*Siglec-15* Sialic acid-binding immunoglobulin-like lectin 15, *PD-L1* programmed cell death-Ligand 1, *WHO* World Health Organization, *D-FFS* distant failure-free survival.

The bold value represents p>0.05, indicating that the difference is statistically significant.

## Discussion

Our study was designed to explore the expression of PD-L1 and Siglec-15 in NPC tumor cells and the effect on the survival time of patients when they are expressed alone or in combination. Our data showed that higher expression levels of PD-L1 and Siglecl-15 were found in patients with poor prognoses, compared to patients with a better prognosis. Therefore, our results showed that PD-L1 and Siglec-15 can act as effective indicators that predict the survival of patients with NPC.

The process and molecular mechanism of PD-1/PD-L1-mediated immune escape during tumor occurrence and development have received significant attention in recent years^23^. The combination of PD-1 and PD-L1, expressed on T lymphocytes and tumor cells, respectively, inhibits T cell activity. This results in a loss of the ability of the T-cells to monitor and kill cancer cells, because of this cancer cells could continue to survive; this is called “immune escape”^24^. PD-L1 is overexpressed in various human cancer cells, and is abundantly expressed in NPC patients diagnosed for the first time^10,25^. When tumor cells overexpress PD-L1, the immune escape induced by the binding of PD-1 to it would result in the maintenance or promotion of malignant biological behavior in tumor cells; subsequently, the prognosis of patients becomes worse^9^. For example, overexpression of PD-L1 indicates that the survival time of patients with malignant melanoma and renal cell carcinoma would be reduced^26,27^. In contrast, overexpression of PD-L1 indicates that the survival time in patients with breast cancer and certain molecular subtypes of non-small cell lung squamous cell carcinoma would be longer^28,29^.

The significance of PD-L1 overexpression in the clinical prognosis of NPC patients has previously been considered to be uncertain and controversial. In our study, we examined the expression of PD-L1 and its related factors in 182 patients with NPC. The results showed that the OS of NPC patients exhibiting PD-L1 overexpression was decreased. Lee et al. stated that PD-L1 overexpression is related to an increased disease-free survival in patients^10^, but this phenomenon has not been observed in this study. The differences in experimental results could be attributed to the lack of a standardized process for quantitative analysis after PD-L1 expression in tissue sections assessed via immunohistochemical staining, the methods for tumor tissue biopsy and sample preparation being different, and the expression of PD-L1 in NPC tissues possibly being affected by heterogeneous changes^30^.

Siglec-15 is known to be expressed on tumor-associated macrophages and promote tumor immunosuppression by increasing TGF-β secretion in conjunction with DAP12 and Syk^21^. Professor Chen’s results suggest that the role of Siglec-15 and PD-L1 in mediating the immune escape of tumor cells is similar, but the role of Siglec-15 in the occurrence and development of cancer is unclear^19^. TCGA database analysis suggested that Siglec-15 mRNAs could be expressed in patients with renal cell carcinoma, lung cancer, colon cancer, and other types of cancer^19^. Immunofluorescence staining analysis results for NSCLC tumor tissues showed that Siglec-15 expression was up-regulated in tumor cells, and further studies have shown that it was expressed at higher levels in lung adenocarcinoma patients than in other types of pathological disease^31^. However, there is a lack of studies on Siglec-15 expression or Siglec-15 mRNA in NPC patients. The results of our study showed that the expression of Siglec-15 was elevated in NPC tumor tissues, and the positive expression rate of tumor cells was 37.9%. In addition, Siglec-15 levels significantly and negatively correlated with PD-L1 levels, which was consistent with the results of Professor Chen’s study, which reported that the expression of PD-L1 and Siglec-15 were mutually exclusive^19^. The results also suggested that patients who could not be treated effectively with the PD-1/PD-L1 monoclonal antibody might benefit from using the Siglec-15 monoclonal antibody^5,32^. At present, treatment with the molecular receptor of Siglec-15 has not been examined. It has been confirmed that the Siglec-15 receptor can bind directly to T cells, but it is not a well-known immune receptor similar to the receptors of the B7 family^33–35^.

Until now, the correlation between Siglec-15 and tumor prognosis has been insignificant. Through pan-cancer analysis, Li et al. have concluded that Siglec-15 overexpression is associated with a poor prognosis, and lung cancer patients exhibiting Siglec-15 overexpression have a significantly decreased progression-free survival period^21^. Fubada et al. have also demonstrated that the overexpression of Siglec-15 is indicative of a good prognosis for patients with primary CNS lymphoma^22^. Our findings showed that Siglec-15 overexpression was related to an increase in the distant metastasis rate of NPC patients, which may be attributable to the fact that Siglec-15-mediated immune escape promotes the malignant biological behavior of tumor cells. Furthermore, our results showed a negative correlation between the expression of Siglec-15 and PD-L1; hence, we further explored whether the combination of PD-L1 and Siglec-15 had an impact on the survival rate of NPC patients. The results for the combined group showed that the distant metastasis rate was higher in the double-positive group and the OS was longer in the double-negative group. We need to identify other potential mechanisms of tumor immune escape apart from those involving Siglec-15 and PD-L1 in further studies.

However, there are still some weaknesses in our study. Previous studies have shown that there is heterogeneity in the expression of PD-L1 in tumors, and that there are differences in the positive rate of PD-L1 between small biopsy and surgical specimens^36^. However, Prof. Melosky compared surgical specimens with small biopsies of lymph nodes for PD-L1 staining. The results suggested that the anastomosis rate of the two was approximately around 90%^37^. However, because of the special location of the tumor in NPC patients, only few cases can choose surgical treatment. Therefore, we chose biopsy specimens, which, although a modality that currently appears acceptable, may have some impact on the final results. More comprehensive studies should be implemented to confirm the results.

In summary, our study confirmed that NPC tumor cells can express Siglec-15 and PD-L1, and that there is a negative correlation between them, but the mechanism for their expression is still unclear. In NPC patients, the positive expression of PD-L1 is a negative independent predictor of decreased OS, and a positive expression of Siglec-15 is a risk factor for distant metastasis. When both Siglec-15 and PD-L1 were not expressed, the OS of NPC patients was significantly prolonged. Thus, these findings provide a reference for predicting the survival and prognosis of NPC patients.

## Conclusion

In conclusion, our study demonstrated that the expression of PD-L1 and Siglec-15 in tumor cells correspond to a poor prognosis in NPC patients. Thus, we predict that PD-L1 and Siglec-15 may be involved in the progression of NPC, and Siglec-15 may serve as another potential promising therapeutic target in NPC treatment. Subsequently, we further investigated the different expression patterns of PD-L1 and Siglec-15 in NPC tumors; a worse prognosis was predicted for NPC patients when they were positive for both these target molecules. Our findings imply that PD-L1 and Siglec-15 might represent novel molecular targets for NPC treatment.

## Materials and methods

### Ethics statement

Data of the patients participating in this study were collected between January 2012 and December 2015. Our experiments were approved by the ethics committee of Southwest Medical University (reference number: KY2021292), and informed consent was obtained from all subjects. Our study was carried out in accordance with the ethical principles of the Declaration of Helsinki.

### Clinical data and treatment

All subjects were rescored according to the seventh edition of UICC/AJCC standards. The enrolled vertebrae were as follows: NPC supported by pathological diagnosis, without any history of surgery, radiotherapy or chemotherapy related to tumor treatment, Karnofsky score of 70; IMRT was performed in the Affiliated Hospital of Southwest Medical University, and traceable regular follow-up data were collected. Patients were excluded if they had an uncontrolled infection; previously received any anti-cancer therapy; were pregnant or lactating mothers; had a previous malignancy, or intolerance to chemotherapy because of vital organ disease.

The basic examination included a combined thoracoabdominal computed tomography scan, magnetic resonance imaging, and fiberscope analysis of the nasopharynx, magnetic resonance imaging of the neck, and bone scan imaging. IMRT treatment was performed in all patients. The treatment strategy of patients included concurrent chemoradiotherapy regimen and induction chemotherapy based on platinum. The induction chemotherapy regimen included 175 mg/m^2^ paclitaxel (day 1) and 75 mg/m^2^ cisplatin (day 1), or 1000 mg/m^2^/day fluorouracil, pumped continuously for 96 h through a micro pump, along with 75 mg/m^2^ cisplatin (day 1). The treatment period was 21 days.

Guidelines of reports 50^38^, 62^39^, 71^40^, and 83^41^, of the ICRU have guided the definition of target volumes in the nasopharynx and nodal regions. The prescribed doses were based on the planned gross tumor volume (GVT) of the nasopharynx and lymph node metastases in the neck, for which regimens were 66–74 Gy/33–35 fractions and 66–70 Gy/33–35 fractions, respectively. For the planned clinical target volume of the high-risk area and low-risk area, the regimens were 63–64 Gy/33 fractions and 53–54 Gy/33 fractions, respectively. The concurrent chemoradiotherapy regimen was the above radiotherapy regimen supplemented with 40 mg/m^2^/w cisplatin, and was administered once daily; a total of five doses were administered in a week.

### Patient follow-up

Follow-up duration was defined as the period from diagnosis to death in confirmed patients or from diagnosis to the last follow-up examination in surviving patients, and the end of follow-up was set to June 2020. As prescribed in our experiments, the frequency of follow-up was every 3 months for the first 2 years after the end of treatment and every 6 months for the next 6 years. The median follow-up for the whole group was specified to be 65 months (range 55 to 101 months). We determined the OS from initial diagnosis to death, FFS from diagnosis to disease failure, LR-FFS and D-FFS from diagnosis to local–regional failure and distant failure separately. Information regarding the duration of survival of patients who were still alive at the time of the last follow-up was censored.

### Immunohistological assays

The obtained specimens were fixed in formaldehyde solution, and fixed specimens were embedded into paraffin wax blocks and cut into 4 μm sections. Sections were subjected to a series of steps, including deparaffinization, hydration, antigen retrieval, immunostaining, and other procedures. Sections were stained with a mouse anti-PD-L1 monoclonal antibody (GB13339; Servicebio, Wuhan, China; 1:400 dilution) based on the standard avidin biotin complex method. Bound antibodies were revealed using a DAB detection kit (G1211; Servicebio). Next, we microscopically visualized membranous PD-L1 expression on tumor cells. PD-L1 ≥ 1% or PD-L1 < 1% on tumor cells was used to define positive and negative expression on cells, respectively. Furthermore, fluorescence immunostaining was used to determine the expression of Siglec-15 in tumor tissues. For fluorescence staining of sections, we used Rabbit anti Siglec-15 polyclonal antibody (ab198684; Abcam, Cambridge, MA, USA; 1:1000 dilution); subsequently, anti-rabbit IgG antibody (GB23303; Servicebio) was added. Analysis was performed using a fluorescence microscope (eclipse C1, Nikon, Osaka, Japan).

### Statistical analyses

The relationship between PD-L1 and Siglec-15 when expressed alone or in combination and clinicopathological variables were assessed with the Chi-squared test. The correlation of expression between PD-L1 and Siglec-15 was assessed with Spearman’s correlation analysis. The Kaplan Meier survival analysis method was used for analyses of cumulative metastasis, cumulative recurrence, and survival probability. The log rank test was used to assess whether the differences were statistically significant. Cox regression method was applied to analyze the prognostic factors related to survival. In all data, a two tailed P-value < 0.05 was defined as a statistically significant difference. All analyses were performed using SPSS 25 software.

## Abbreviations

NPC: Nasopharyngeal carcinoma
Siglec-15: Sialic acid-binding immunoglobulin-like lectin 15
PD-L1: Programmed death-ligand 1
NSCLC: Non-small cell lung cancer
IMRT: Intensity-modulated radiotherapy
IgV: Immunoglobulin variable region
IgC2: Type 2 constant region
UICC/AJCC: Union for International Cancer Control/American Joint Committee on Cancer
OS: Overall survival
FFS: Failure-free survival
D-FFS: Distant failure-free survival
LR-FFS: Local regional failure-free survival
